# Catheterization laboratories open the doors for Extended Realities—review of clinical applications in cardiology

**DOI:** 10.1093/ehjdh/ztaf072

**Published:** 2025-06-23

**Authors:** Maria Kundzierewicz, Katarzyna Kołodziej, Arif Khokhar, Tsai Tsung-Ying, Artur Leśniak, Pawel Zakrzewski, Hubert Borecki, Ewelina Bohn, Jan Hecko, Jaroslav Januska, Daniel Precek, Maciej Stanuch, Andrzej Skalski, Yoshinobu Onuma, Patrick Serruys, Nico Bruining, Adriana Złahoda-Huzior, Dariusz Dudek

**Affiliations:** SimHub, Virmed, Kraków, Poland; SimHub, Virmed, Kraków, Poland; The Heart Center, Rigshospitalet, Copenhagen University Hospital, Copenhagen, Denmark; CORRIB Research Centre for Advanced Imaging and Core Laboratory, National University of Galway, Galway, Ireland; SimHub, Virmed, Kraków, Poland; SimHub, Virmed, Kraków, Poland; SimHub, Virmed, Kraków, Poland; SimHub, Virmed, Kraków, Poland; Cardiocentrum Trinec, Agel, Trinec, Czech Republic; Faculty of Electrical Engineering and Computer Science, VSB - Technical University of Ostrava, Ostrava, Czech Republic; Cardiocentrum Trinec, Agel, Trinec, Czech Republic; Cardiocentrum Trinec, Agel, Trinec, Czech Republic; Department of Measurement and Electronics, AGH University of Krakow, Al. Adama Mickiewicza 30, Krakow 30-059, Poland; MedApp S.A., Krakow, Poland; Department of Measurement and Electronics, AGH University of Krakow, Al. Adama Mickiewicza 30, Krakow 30-059, Poland; MedApp S.A., Krakow, Poland; CORRIB Research Centre for Advanced Imaging and Core Laboratory, National University of Galway, Galway, Ireland; CORRIB Research Centre for Advanced Imaging and Core Laboratory, National University of Galway, Galway, Ireland; Department of Cardiology, Thoraxcenter, Erasmus MC Rotterdam, Rotterdam, Netherlands; SimHub, Virmed, Kraków, Poland; Department of Measurement and Electronics, AGH University of Krakow, Al. Adama Mickiewicza 30, Krakow 30-059, Poland; Center for Digital Medicine and Robotics, Jagiellonian University Medical College, Krakow, Poland; GVM Care & Research, Maria Cecilia Hospital, Cotignola, Italy

**Keywords:** Extended Realities, Virtual reality, Augmented reality, Mixed reality, 3D imaging, Cardiac imaging, Pre-procedural planning, Intra-procedural navigation

## Abstract

The complexity and spatial relationships between vascular and cardiac structures, as well as anatomical diversity, pose a challenge for planning and performing cardiac interventions. Medical imaging, especially precise three-dimensional imaging techniques, plays a key role in the decision-making process. While traditional imaging methods like angiography, echocardiography, computed tomography, and magnetic resonance imaging remain gold standards, they have limitations in representing spatial relationships effectively. To overcome these limitations, advanced techniques such as three-dimensional printing, three-dimensional modelling, and Extended Realities are needed. Focusing on Extended Realities, their main advantages are direct spatial visualization based on medical data, interaction with objects, and immersion in cardiac anatomy. These benefits impact procedural planning and intra-procedural navigation. The following publication presents current applications, benefits, drawbacks, and limitations of Virtual, Augmented, and Mixed Reality technologies in cardiac interventions. The aim of this review is to improve understanding and utilization of the entire spectrum of these innovative tools in clinical practice.

## Graphical Abstract

**Graphical Abstract:**
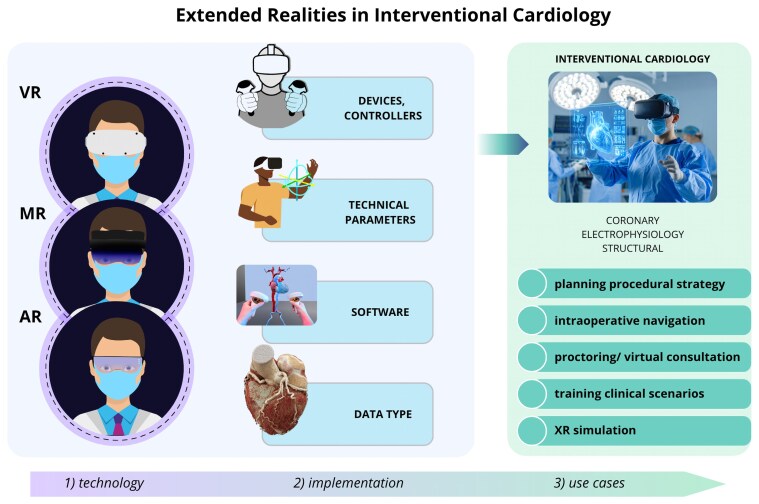

## Introduction

Vascular and heart structures consist of complex three-dimensional (3D) systems. Planning and performing procedures in these areas is difficult due to significant anatomical variability. A key element in planning and performing cardiac procedures is precise medical imaging that encapsulates structures, flows, and morphology. In recent years, 3D visualization of cardiac structures has become increasingly important. 3D imaging allows physicians to guide the intervention with specific tool selection. The knowledge and experience of radiologists, interventional cardiologists, and cardiac surgeons, among others, are essential for the successful planning of cardiac treatment strategy.^1^

Invasive coronary angiography (ICA), echocardiography, computed tomography angiography (CTA), and magnetic resonance (MRI) are currently the gold standard of cardiac imaging. Planning is mainly based on two-dimensional (2D) visualization of the patient’s heart anatomy. Increasing complexity of interventions requires more advanced imaging techniques for better recognition of the spatial location of the patient’s intracardiac and extracardiac structures.^2^ Depending on the procedure, the appropriate imaging method should be selected. CTA is used to assess the entire vessel and visualize the coronary anatomy in 360°. Echocardiography is most often performed to assess the morphology and function of the heart valves and to detect intravascular structural anomalies. MRI is mainly utilized for visualizing cardiac function, while ICA is used for intra-procedural guidance.^3^

The current way of 3D anatomical structures presentation on flat 2D screens impedes depth and spatial relationship perception. This can lead to errors in image interpretation. Hence, it is important to have extensive knowledge of spatial orientation and imagination in order to effectively interpret these images.^2,3^

Visualization technologies that can help overcome the errors associated with two-dimensional imaging include 3D printing, 3D modelling, and Extended Realities (XR). 3D printing allows for the physical manipulation of anatomical models but does not incorporate hemodynamic features. In contrast, 3D modelling allows for precise measurements but remains confined to a two-dimensional display on a computer monitor.^3,4^

**Figure 1:**
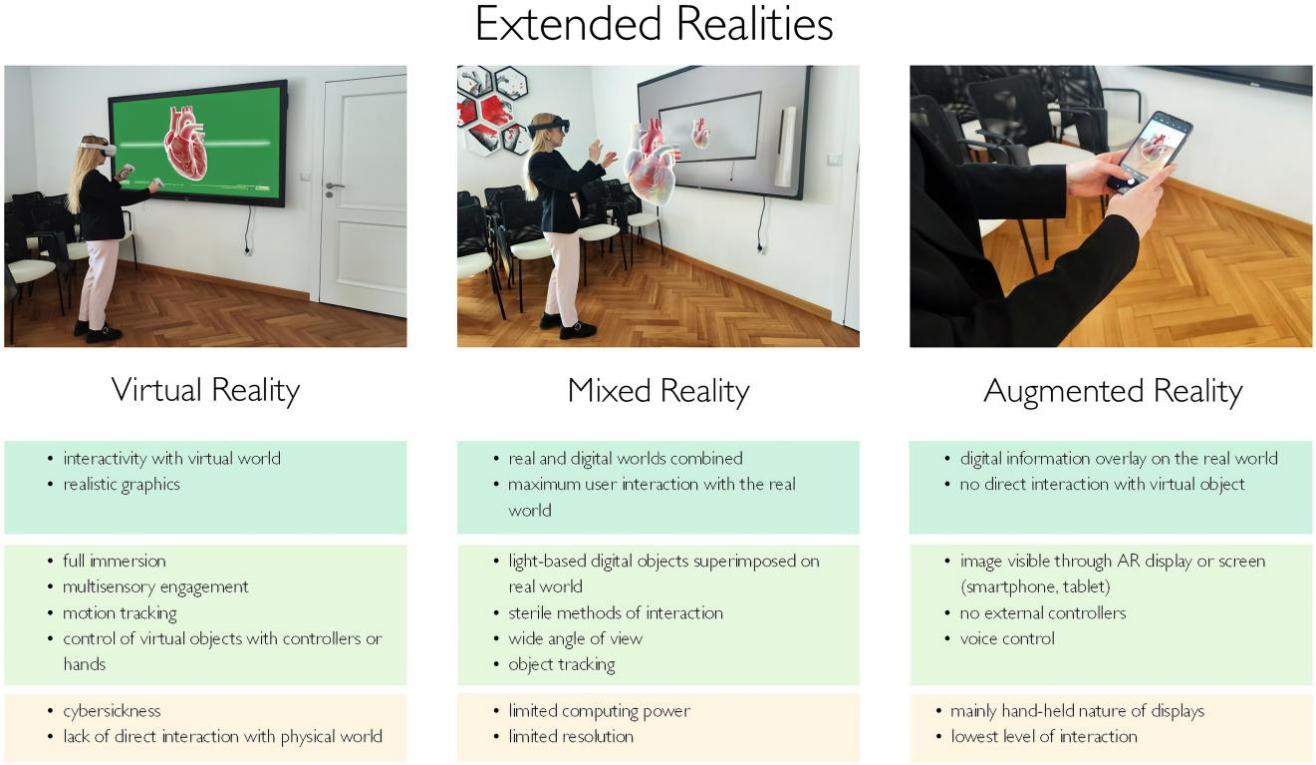
Spectrum of Extended Realities (XR): Virtual (VR), Mixed (MR), and Augmented Reality (AR) with their main features.

XR refer to those visualization techniques that generate a combined—physical and virtual—reality and include subordinate techniques such as Virtual Reality (VR), Augmented Reality (AR), and Mixed Reality (MR) (*Figure 1*).^5^ XR have some unique advantages over 3D printing and 3D modelling. The visualization of the anatomy is detailed, interactive, and not limited to a single cut plane or viewing window as with a physical model. Using XR, all relevant structures can be reproduced with the highest fidelity, with the added benefit of being able to scale in size according to operator preference.^4^ In order to combine virtual and physical worlds, the XR headsets use SLAM (Simultaneous Localization and Mapping) algorithms, responsible for the device’s understanding of position, orientation, and manipulation in the physical environment. As a result, 3D reconstructions or gesture recognition are correctly interpreted and ensure that virtual elements match the real world.^6^

This article provides an overview of current applications of Virtual, Augmented, and Mixed Reality technologies in the planning and performance of cardiac procedures, including coronary, electrophysiology, and structural interventions performed either surgically or percutaneously. We compare commercially available VR, AR, and MR devices and softwares, highlighting their specifications and average pricing. The article also examines the clinical applications of these technologies, describing the benefits of using XR-based medical imaging in both pre-procedural planning and intraoperative navigation. Furthermore, it identifies the limitations and challenges associated with the implementation of these technologies. Based on the compiled database, the review also provides insights and recommendations for future research directions.

## Virtual, augmented, mixed reality

### Virtual reality

Virtual Reality (*Figure 1*) is a technology which refers to a computer-generated 3D environment, allowing users to interact with digital realities. Virtual images imitate real, fictional, or mixed spaces, objects, and incidents.^7^ Visualizations are presented separately (with a slight shift) to the right and left eye. Once they are superimposed, the brain creates a 3D image. To make the effect of VR as realistic as possible, the images are displayed in VR headsets impermeable to the real environment visually. Currently, multiple VR displays are commercially available. Depending on user requirements and budget, several factors should be considered when selecting a device (*Tables 1* and *2*). Models with lower resolution and refresh rate may impact the virtual environment fluency. For auditory sensations, VR devices are equipped with dedicated headphones and microphones. Furthermore, they offer users the capability of virtual connections irrespective of a user’s physical locations. In addition to visual and auditory experiences, sensory immersion is equally important. Feedback or other physical sensations, such as vibrations generated by VR headsets or joysticks can increase a user’s emotional and physical engagement, making virtual experiences more tangible and real.^8^

**Table 1:** Extended realities devices—technical specification

Device	Specifications						
	RAM	Storage	Field of view	Refresh rate	Battery life	Weight	Display resolution
VR							
Oculus Quest (2019)	4 GB	64/128 GB	100°	72 Hz	2–3 h Active use	571g	1440 × 1600 Pixels per eye
Oculus Quest 2 (2020)	6 GB	64/128/256 GB	89° (+/− 4°) Both horizontally and vertically	90 Hz	2–3 h Active use	503g	1832 × 1920 Pixels per eye
Oculus Meta Quest Pro (2022)	12 GB	256 GB	106° Horizontal × 96° vertical	90 Hz	2–3 h Active use	722g	1800 × 1920 Pixels per eye
Oculus Meta Quest 3^a^ (2023)	8 GB	128/512 GB	110°	90 Hz	2,2 h Active use	515g	2064 × 2208 Pixels per eye
VIVE XR Elite (2023)	12 GB	128 GB	110°	90 Hz	up to 2 h	273g	1920 × 1920 Pixels per eye (3840 × 1920 pixels combined)
Apple Vision Pro^a^	16 GB	256/512 GB 1 TB	100°	90 Hz, 96 Hz, 100Hz	Up to 2 h of general use	600–650g	3660 × 3200 Pixels per eye
VIVE Pro 2 (2021)	16 GB	64 GB	Up to 120°	90/120 Hz	Wire	850g	2448 × 2448 Pixels per eye
VIVE Cosmos (2019)	8 GB	no data available	Up to 110°	90 Hz	Wire	645g	1440 × 1700 pixels per eye
VIVE	✘	✘	Up to 110°	90 Hz	Wire	440g	1080 × 1200 Pixels per eye
AR							
Google Glass 3.0 (2017)	2GB	16 GB (12 GB available)	No data	60/70 Hz	570 mAh	50g	640 × 360 Pixels
Samsung Galaxy S24 Ultra^b^	8/12GB	128/256/512GB	110–120°	120 Hz	4000–5000mAh	150–200g	2560 × 1440 Pixels
Glass Enterprise Edition 2 (2019)	3GB	32 GB	83° Diagonal field of view	90 Hz	800 mAh	46g	640 × 360 Pixels
Apple iPhone 15 Pro Max^b^	8/12GB	256/512/1000GB	110–120°	120 Hz	4422 mAh	221g	2796 × 1290 Pixels
MR							
HoloLens (2016) United States	2 GB	64GB	30° Horizontally 17.5° vertically	60 Hz	2–3 h	579g	1280 × 720 Pixels per eye
HoloLens 2 (2019) United States	4 GB	64GB	50° Horizontally 40° Vertically	75 Hz	2–3 h	566g	2048 × 1080 Pixels per eye
Magic Leap One (2018) United States	8 GB	95GB	40° Horizontally 45° vertically	120 Hz	3 h	345g	1160 × 1280 Pixels per eye
HOLOSCOPE-i (2017) – medical only	No data	No data	Full visibility of surroundings	No data	Several hours—depending on the intensity of use	No data	No data

^a^Headset can be defined as Virtual or Augmented Reality based on possibility to present the real environment with camera's support.

^b^Any smartphone can be used as an AR device, the table includes top examples of the devices running on different operational systems—Android and iOS.

**Table 2:** Extended realities devices—additional practical information

Device	Software	Connectivity			Controllers/Sensors			Price
		USB	Bluetooth	WiFi	Handheld motion controllers	Eye-tracking	voice commands	
VR								
Oculus Quest (2019)	Oculus Quest system software, based on Android source code Qualcomm Snapdragon 835	C	✓	✓	✓	✘	✘	Up to 550 USD
Oculus Quest 2 (2020)	Oculus Qualcomm Snapdragon XR2 Platform	C, C3	✓	✓	✓	✓	✓	250–600 USD
Oculus Meta Quest Pro (2022)	Oculus Qualcomm Snapdragon 662	C	✓	✓	✓	✓	✓	1000–1750 USD
Oculus Meta Quest 3 (2023)	Oculus Qualcomm Snapdragon XR2 Gen. 2	C	✓	✓	✓	✓	✓	500–1000 USD
VIVE XR Elite (2023)	HTC VIVE Qualcomm® Snapdragon™ XR2	3.2 Gen-1, Type C	✓	✓	✓	✓	No data	Up to 1760 USD
Apple Vision Pro	Apple Vision	C	✓	✓	✓	✓	✓	6300 USD
VIVE Pro 2 (2021)	HTC VIVE	C	✓	✘	✓	✓	✓	Up to 1000 USD
VIVE Cosmos (2019)	HTC VIVE and Steam VR software	C	✘	✘	✓	✘	✓	Up to 750 USD
VIVE	HTC VIVE software	3.0 others: HDMI, mini display port	✘	✘	✓	✘	No data	No data
AR								
Google Glass 3.0 (2017)	Google Android	Micro	✓	✓	*Voice command:* only English *Touch buttons*: on the right side on the top *Eye sensors:* respond to wink *User interface:* side-mounted touchpad			1800 USD
Samsung Galaxy S24 Ultra	Android 14	Type C	✓	✓	*User interface:* Multi-touch gesture touchpad			1800 USD
Glass Enterprise Edition 2 (2019)	Google SoC—Qualcomm Snapdragon XR1 OS—Android Open Source Project 8.1 (Oreo)	2.0, Type C	✓	✓	*User interface:* Multi-touch gesture touchpad			1000 USD
Apple iPhone 15 Pro Max	iOS 17	Type C	✓	✓	*User interface:* Multi-touch gesture touchpad			2200 USD
MR								
HoloLens (2016) United States	Microsoft Windows 10, Windows Mixed-Reality	2.0	✓	✓	Spatial sound Gaze tracking Gesture input Voice support			3000 USD
HoloLens 2 (2019) United States	Microsoft Windows Holographic Operating System Microsoft Edge Dynamics 365 Remote Assist, 3D Viewer	C	✓	✓	Hand tracking Eye real-time tracking Head tracking Voice Command			3500 USD
Magic Leap One (2018) United States	Magic Leap Qualcomm Snapdragon 835	C	✓	✓	Spatial Audio Device support 6 DoF tracking of the headset, hands and controller, Displays are made with the help of the Virtual retinal monitor technology			2300 USD
HOLOSCOPE-i (2017) – medical only	RealView	Not given	Not given	Not given	Voice, gaze, and gestures—touch-free input			No data

To create a full perception of reality in VR, it is important to keep positional tracking both accurate and precise. Interactive influence on the displayed reality is possible with controllers, pads, and joysticks, which transfer user actions to the digital space—from simple button presses to advanced hand tracking for precise interaction with virtual objects. Several methods use sensors to record signals from transmitters, sending data to a computer or cloud to approximate locations. Additionally, gyroscopes and accelerometers built into the head-mounted displays (HMD) can determine head movements and gaze direction in real time.

An undesirable effect of using VR devices is cybersickness, a type of motion sickness generated by a shifted view in a headset or a view incompatible with audio sensation. Due to cybersickness, extended use of VR technology can lead to loss of spatial awareness, disorientation, and other disorders.^9^ To avoid emerging issues related to cybersickness, regular device use with user-specific calibration may be helpful. Reducing the weight of VR devices, parallel with their technical development (eye-tracking, higher refresh rate, and haptic feedback) may positively impact visual distortions.

### Augmented reality

Augmented Reality (*Figure 1*) combines real environments with digital images, allowing for the simultaneous presentation of both real and virtual objects. Importantly, the interaction with virtual objects is indirect, and digital images are visible only when the user looks directly through the device equipped with the AR software. Specifically, there are three main categories of display devices that have an important position in the field of AR technology: HMD (specially dedicated glasses), handheld device displays (smartphones, tablets), and other display devices, such as a PC desktop display. Unlike VR glasses, AR devices allow external light to pass through. As a result, they do not cut the user off from the real world. In the AR systems, 3D registration technology enables virtual images to be superimposed onto a real environment. 3D registration might be performed using either automatic or manually set reference points.^10^ To achieve high-resolution, fluent, and precise 3D imaging, it is important to choose an appropriate AR display that meets the technological and quality requirements. Devices using AR technology often don’t need any external controllers; however, there are some exceptions. Google Glass, for instance, uses a touchpad located on the side of the glasses which allows users to control the device and displayed images. In some other devices, mounted microphones allow for the presented image to be controlled by voice commands. Additional sensors and inputs such as GPS, gyroscopes, accelerometers, and compasses can further raise the quality of the observed digital image superimposed on the real environment.^10,11^

A practical advantage of AR technology displays is their low weight compared to VR devices. However, some of those devices can be uncomfortable to use due to their size (tablets and computer screens).

### Mixed reality

Mixed Reality (*Figure 1*) provides users with the greatest interaction between the real-world and the digital world compared to other XR technologies. This allows for a more natural and intuitive interaction between multiple users, as well as both the real and virtual environments. A feature of MR is the ability to place a digital object, in the form of a hologram (object made from light, usually combined with sound), in the real world as if it was physically present.^12^ The method of interaction is mainly sterile and occurs through voice commands, gestures, gaze, or digital panels displayed in front of the user in a holographic form. In the most intuitive form, users can play with objects using their hands. With newer generation devices, each finger movement is recognized separately in order to naturally mimic the interactions with a physical object.

MR Systems are divided into optical see-through and video see-through systems. In optical see-through systems, users can see the real world directly with the virtual objects added. Video see-through systems use cameras to capture the surrounding environment, which is processed and reprojected as video with MR objects mapped.

Importantly, issues such as induced distortion, latency, refresh rate, colour representation, and resolution may lead to users experiencing unsynchronized, blurry, or unnatural view.^13^

A big advantage of MR systems is the use of all 6° of free movement and the wide viewing angle (up to 120°), which allows the user to freely move around holographic objects, instead of looking from a fixed point of view.^14^ On the other hand, two main limitations of MR systems are: holographic display and tracking. The MR platform should be based on an appropriate screen technology, such as OLED (Organic Light-Emitting Diode) or microLED. OLED allows for deep contrast and vibrant colours, while microLED is recommended for higher brightness and energy efficiency. Interaction between virtual and real objects requires precise methods to track both, to make the final impression realistic.^15,16^

## Devices

**Figure 2:**
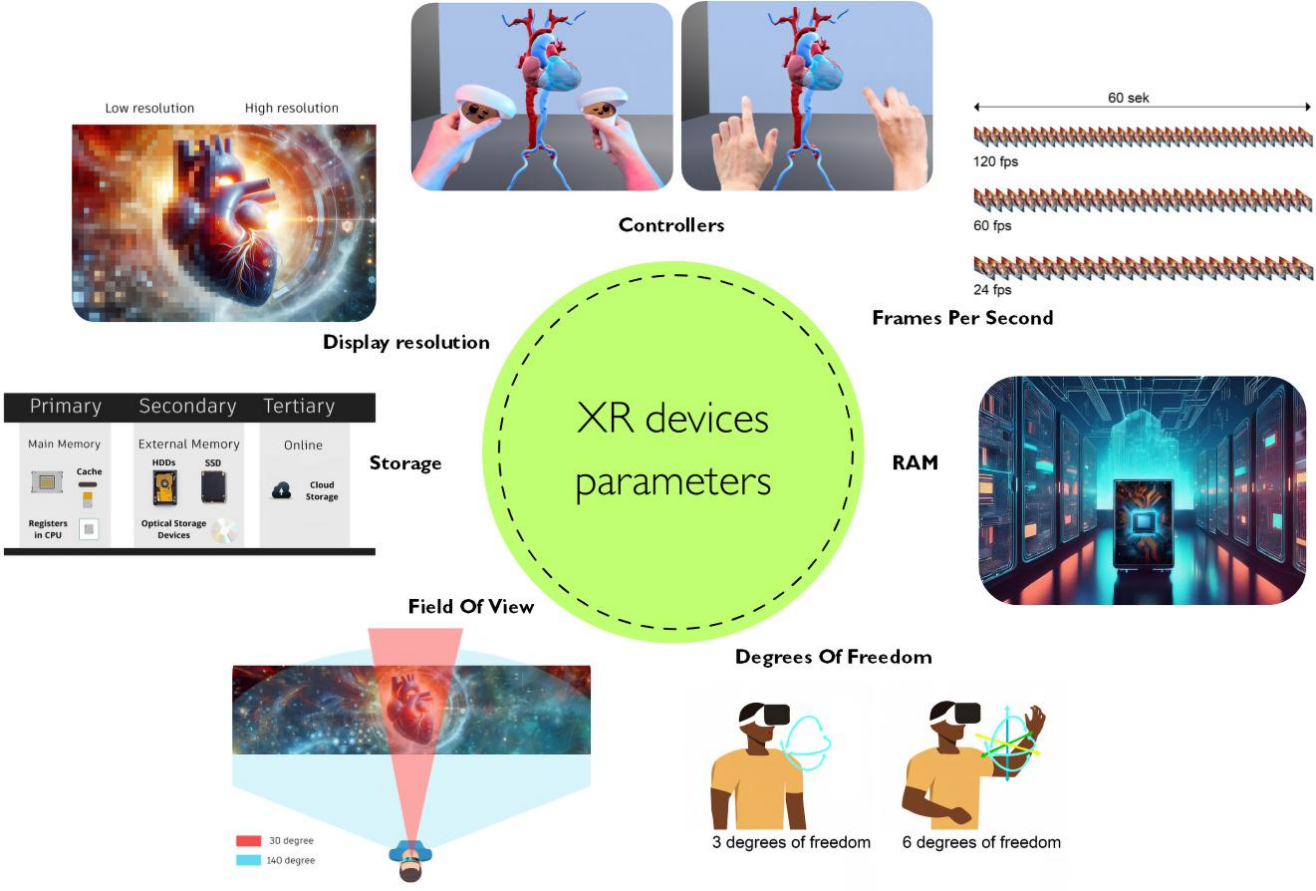
Graphical explanation of main technical parameter differences among different XR technologies.

XR Technology nowadays features compact, mostly wireless devices. *Tables 1* and *2* summarize the devices’ specifications, including connectivity, memory parameters, processing power, and battery longevity. In addition, the small size and weight of the devices do not exclude the high quality and resolution of the displayed images suitable for clinical settings. When evaluating the currently available devices, the following parameters should be considered (*Figure 2*).

### Field of view

Field of view (FoV) defines the area seen by the XR user. Setting the FoV value to 90° allows the user to see an image of the 90° range in an instant. Hence, the larger FoV the device supports, the more virtual reality the user can see around. Therefore, in order to ensure the most natural experience of using XR, the value of the FoV parameter should be as close as possible to the real value of the FoV of the human eye (approximately 200°).^17,18^

### Refresh rate

The screen refresh rate defines how many times in one second the device is able to generate a new image. The higher the frame rate, the more image frames can be displayed, which improves the image fluency and reduces the blur effects. The refresh rate is affected by the source image quality, the processing power of the device, and the display itself. An important parameter that is related to the refresh rate is frames per second (FPS). Smooth motion display is described as the number of frames rendered every second. Image processing by the human eye is subjective and depends on the characteristics of the visual stimulus. The refresh rate threshold at which a human considers the image as smooth is assumed to be 50 Hz.^18^ As the refresh rate increases, the smoothness and realism of virtual reality images increase as well.^17^

### Display resolution

Display resolution determines the number of pixels displayed on the HMD screen, affecting the sharpness of the observed image. Display resolution is usually given in pixels per eye or radian. The greater the number of pixels (greater display resolution value), the better and clearer image can be achieved. Increasing the resolution impacts higher demand for the computing power of the device (greater demand for energy). Additionally, the ability to provide enough luminosity against a bright background (e.g. surgical theatre) and the faithfulness of colour should also be considered.^19^

### Controllers

Simple XR controllers with only classic buttons offer selection, activation, or trigger specific digital items. More advanced sets are able to precisely transfer hand or even finger movements directly into the virtual environment. Eye control and voice commands provide additional support, making the XR experience closer to reality, as they mimic real-world behaviours like looking and speaking.^20^

### Degrees of freedom

Degrees of freedom (DoF) define the state of the physical system, most often body movement in space. The more advanced the XR device is, the more DoF it can handle. Older devices support only three levels (3-DoF), covering head movements like nodding, twisting, and rotating. The HMD with 6 DoF allows the user also to move forward, backward, side to side, as well as squat and rise, providing more movement flexibility.^21^

### Storage

Storage is any type of computing hardware used to store all applications, operating system, and digital content (photos, videos, and documents) for an indefinite period of time. Storage devices can store data temporarily and permanently.^20^

### Computing power

Computing power refers to the ability of a computer system to process data and perform tasks. In other words, it is the ability of a device to process data to obtain a specific result. It relies on computing resources such as the central processing unit (CPU), graphics processing unit (GPU), and random access memory (RAM).

The CPU processes data from sensors, cameras, and user inputs like gestures and voice command devices to create a coherent and interactive XR experience. The GPU is dedicated to rendering images including textures and animations in XR devices for smooth and high-quality experiences.

RAM affects the speed of the computer, so the greater the memory is, the faster the processing speed. The size of RAM determines how much data can fit into memory. Most of the graphics processing units in HMD are not capable of visualizing medical data using volume rendering due to limited power resources. That is why, some applications are computer- or cloud-based solutions, based on computing power available on the server device.^22^

## Extended Realities softwares

**Figure 3:**
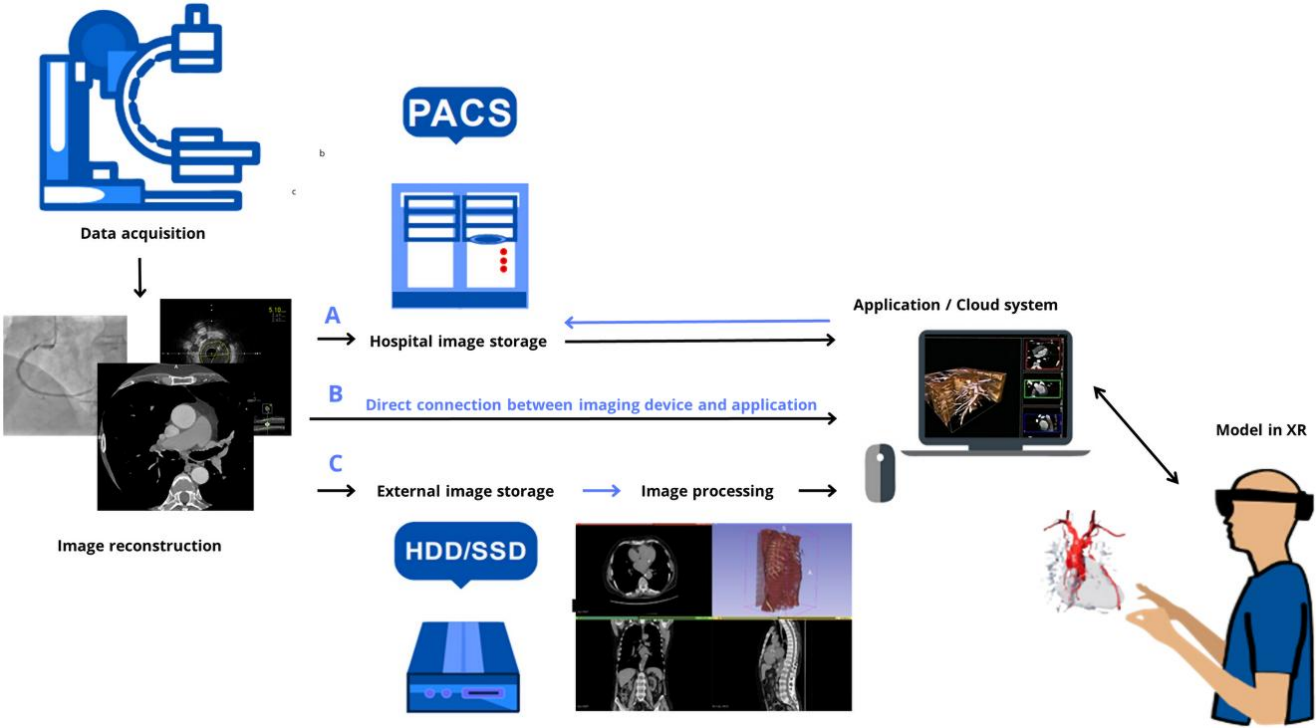
Clinical workflow of Extended Realities (XR) data visualization. Post data acquisition, images are reconstructed and transferred either to image storage or directly—to XR application. The application allows users to visualize and interact with the 3D virtual objects via head-mounted displays and dedicated controllers.

Each device amongst the XR technologies (*Figure 3*) requires appropriate software to enable medical use, and can vary significantly in their capabilities.^23^

Basic XR softwares allow users to view virtual images in 3D, whereas more advanced ones offer interaction features such as rotation, scaling, and movement of displayed images (AnatomyX, Medivis).

Numerous softwares enable users to access data from the Digital Imaging and Communications in Medicine (DICOM) system on XR devices. Some of these softwares, like TeraRecon, can be integrated with hospital patient archiving and communication systems (PACS) to synchronize 2D and 3D views for side-by-side comparisons. MR-specific softwares such as OpenSight (Novard) utilize cameras and sensors to map both the patient and the environment, ensuring precise image registration. Some softwares like SentiAR ELVIS (The Enhanced Electrophysiology Visualization and Interaction System), which aids in visualizing anatomy during electrophysiological mapping and ablations, allow real-time data sharing among users in the same room. For procedure guidance, certain softwares offer functionality to navigate anatomical structures using voice commands or gestures without compromising sterility. Direct connection between imaging devices and XR software allows intraoperative support with real-time image sharing, as seen in CarnaLife Holo (MedApp).

The number of clinical softwares available for education, preoperative planning, rehabilitation, and intraoperative visualization is significant.^24^ XR Technologies are also used for patient sedation during procedures, offering an immersive experience to reduce anxiety and improving patient cooperation and the overall experience. In Supplementary material online, *Table S1*, we present the applicability of XR softwares in cardiology practice, focusing on their use in procedural planning as well as intraoperative visualization.

Among XR softwares, VR is mainly used for the development, evaluation, and implementation of immersive procedure planning systems. VR Provides complete control over visualization shown to the operator, ensuring appropriate image presentation.^24,25^ Choosing this technology for surgical planning is based on features such as intuitive interaction with data as well as a full set of integrated measurement and manipulation tools like rotation, zooming in/out, and cropping the image.

AR technology is used to provide relevant information to the user in specific situations. The fusion of different digital images into a real scene in real-time can be useful for intraoperative visualizations. AR softwares include mapping systems and analytical tools to collect and process data during surgery, as well as recording and transmitting functions for remote consultations. AR softwares also allow direct control of the display, without compromising sterility.^23^

MR softwares, equipped with data analysis or object recognition tools, are a convenient technology, which can be utilized during operations. The size, colour transfer function, or viewing angle of a holographic object can be intuitively modified, and once virtual markers are applied to the patient’s body, a precise schema for the procedure can be achieved.^26^ Interactive MR devices with next-generation computing power can be directly connected with PACS systems, which offers direct access to patient imaging data storage. MR softwares also allow for remote intraoperative navigation, simulation, and guidance.

## Clinical applications of Extended Realities

**Figure 4:**
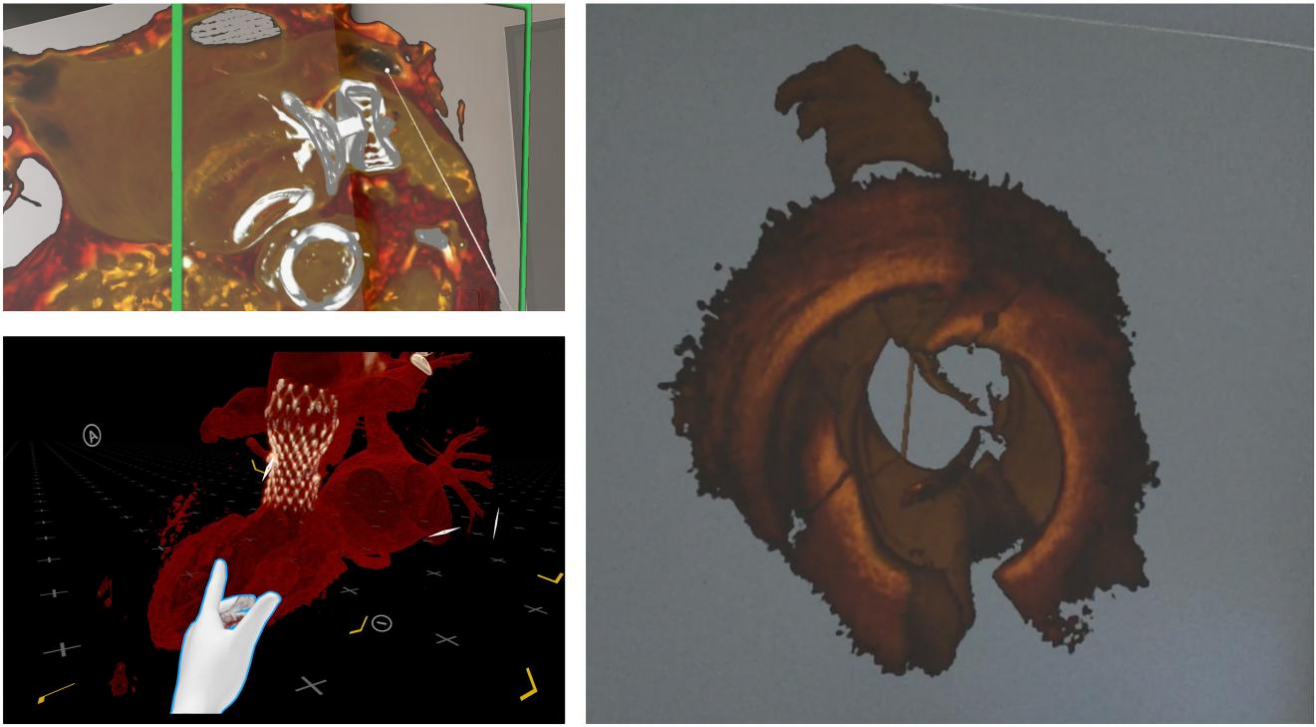
Selected example of pre-procedural and postprocedural use of Extended Realities (XR). (*A*) Evaluation of occluder implantation position post left atrial appendage closure in Mixed Reality using computed tomography images. The view presents left atrial and left atrial appendage cut longitudinally. (*B*) Result of transcatheter aortic heart valve implantation in Virtual Reality (VR). Valve frame implantation height is presented in the aortic root and left ventricular outflow tract, including the left ventricle blood pool in visualization. (*C*) Procedural planning based on Mixed Reality-based optical coherence tomography (OCT) data visualization. Vessel dissection shape and distribution are presented to the operator to plan the stenting strategy and perform spatial lumen measurements.

XR Technologies are increasingly being applied in cardiac interventions to visualize data for planning and navigate coronary, structural, and electrophysiological procedures (see Supplementary material online, *Table S1*, Supplementary material online, *Table S2,*  *Figure 4*).

### Literature research

A comprehensive literature review was conducted to analyse scientific articles and case reports on the use of XR solutions in cardiac procedures. The search was conducted using Google Scholar and Pubmed databases. Research was limited to studies focusing on pre-procedural planning and intra-procedural navigation supported by XR technologies. Studies related to rehabilitation, patients experience, and educational applications were excluded.

The search strategy included a diverse combination of the following keywords: ‘Virtual Reality’, ‘Augmented Reality’, ‘Mixed Reality’, ‘Extended Reality/ies’, ‘holography’ ‘cardiac imaging’, ‘cardiology’, ‘cardiac interventions’, ‘cardiac surgery’, ‘pre-procedural planning’ and ‘intra-procedural navigation’ among other related terms. The research was limited to English-language articles.

The review included studies published till January 2025. In addition, reference lists of selected articles were analyzed to identify further relevant studies.

### Coronary interventions

Coronary interventions are directed at improving blood flow through the coronary arteries, which supply the heart with blood and oxygen. Multiple studies have been described where the XR has been utilized for planning and performance of cardiac procedures (see Supplementary material online, *Table S1*, Supplementary material online, *Table S2*) such as minimally invasive coronary artery bypass surgery^27^ and percutaneous coronary interventions (PCI).^28,29^ In all cases XR technologies were used to plan the intervention and visualizations were performed based on CT images. In some studies, preoperative XR imaging offered a detailed understanding of anatomical structures, allowing users to have an immersive 360° overview^27,28^ to evaluate complex cases. Virtual consultation with other experts^29^ and simulation of guiding catheter selection^28^ have been shown to be important features. In intraoperative navigation in coronary interventions (see Supplementary material online, *Table S2*), XR were used for PCI, especially chronic total occlusion interventions. MR and AR modalities based on CT were adopted to select projections or perform anatomical measurements.^30^ XR visualizations allowed the presentation of the distal segment of the occluded vessel and verification of the guidewire advancement direction.^31,32^ Another application was remote monitoring during the procedure.^33^ The highlighted studies were either single case studies^27,29–31^ or single-site studies with small patient samples.^29,32,33^

### Electrophysiology interventions

Electrophysiological procedures include the diagnosis and treatment of cardiac dysrhythmias, such as atrial fibrillation, tachycardia or conduction blocks, using cardioversion, ablation, and implantation of electronic devices. In the presented cases of planning electrophysiological procedures (see Supplementary material online, *Table S1*), only VR visualizations based on CT images were used. One study was a single case,^34^ while one was a single center and referred to a small group of patients.^35^ VR was used to reconstruct the anatomy and track the access path of the guiding catheter before a difficult ablation procedure.^34^ Preoperative use of VR technology also concerns preparation for the removal of the cardiac electronic system by transvenous wire extraction and the technical approach to this procedure.^35^ For the application of intraoperative navigation in electrophysiological procedures (see Supplementary material online, *Table S2*), MR or AR technologies with CT imaging were selected. The described publications concerned single cases^36,37^ or single-center studies on a small sample of patients.^38–40^ XR have been used for intraoperative navigation in cases with complex patient anatomy. XR provided additional insights into cardiac anatomy and was used to define fluoroscopy angles during pacemaker insertion.^37^ CTA data was processed in real time using voice prompts. During cardiac resynchronization therapy, the operator used XR to assess venous anatomy and select vascular access sites by overlaying holograms.^36^ In relation to transcatheter cardiac mapping and ablation, XR technology provided visualization of presented 2D images in a 3D stereoscopic display.^39,40^

### Structural interventions

Structural procedures include closure of ventricular or atrial septal defects and treatment of heart valve disease with transcatheter or surgical techniques. Among the cited studies (see Supplementary material online, *Table S1* and Supplementary material online, *Table S2*), a spectrum of VR, AR, and MR technologies was used for procedure planning, while AR and MR were chosen for intraoperative navigation. The input data for virtual visualizations were CT, MRI, angiography, echocardiography, or ultrasonography. Structural procedure planning was performed in single-case^41–47^ or single-center studies.^48–68^ In contrast, publications on intraoperative navigation during structural procedures refer to first-in-man experiences^43–72^ or single-center studies^73–77^ with small patient samples. 3D Imaging was mainly used to understand valve position^65–67^ in intra-procedural navigation. XR visualizations were applied in difficult and complex cases planning, as a complement to standard modalities.^41,51,57,63,66^ The 3D review of structures and their relationships was used as a tool to support procedural strategies and influence surgical decisions.^59^ Three-dimensional measurements were performed to influence the selection of implantable devices.^50,58,62,64^ For intraoperative navigation in structural procedures, the XR modalities were used to extend the operator’s field of view.^72,74^ Holographic visualizations were rotated, zoomed, and cropped using only voice commands, providing additional views translated into the positioning of instruments^71^ and management of clinical scenarios.

## Extended Realities combined with different visualization technologies

**Figure 5:**
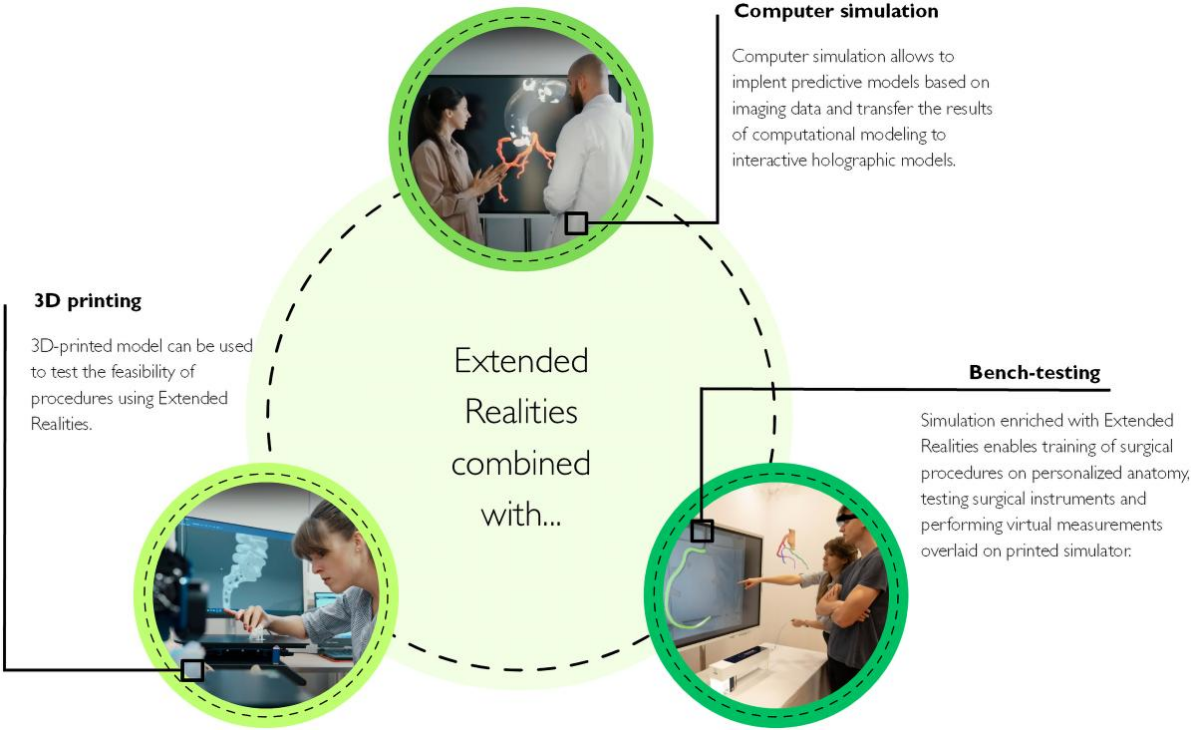
Possibilities of combining Extended Realities (XR) with other technologies—the benefits that can be obtained.

XR can be combined with various technologies to develop more comprehensive and effective diagnosis, planning, and treatment tools for cardiology (*Figure 5*, Supplementary material online, *Table S3*).

3D printing itself allows physical manipulation and direct insight into the anatomical area of interest. 3D printing technology plays a crucial role in diagnosis, surgery planning, and creating custom solutions for patients with heart disease. Using 3D-printed models minimizes the risk of procedural complications and enables to precisely develop a surgical strategy. However, the static nature of the model makes it difficult to realistically represent the physiology of the cardiovascular system. 3D Printing itself does not have hemodynamic properties; however, it is possible to integrate it into a full high-fidelity simulation, which contains a physical simulator plugged with a pulsatile pump to mimic real blood flow. As the solution might be expensive and time-consuming, it is not purely dedicated to daily use.

3D Printing combined with XR and computer or physical simulation creates more interactive and immersive environments for doctors and patients (see Supplementary material online, *Table S3*). Through the use of XR, physicians have the ability to superimpose the actual image of patient heart anatomy on 3D-printed models or high-fidelity physical simulators. With the digital XR tools, it is possible to simulate procedures in XR^78^ or in 3D printing^79^ before they are actually performed. This allows for a precise comparison between the model and the patient’s actual condition, which significantly facilitates the identification and analysis of defects or lesions, and makes it possible to determine the optimal treatment strategy. Moreover, XR simulation has the potential to solve complex clinical scenarios, such as difficulties related to catheter access to coronary ostia.

Integrating holographic instructions with physical and computer simulation can support educational processes for performing cardiology procedures or testing surgical instruments (see Supplementary material online, *Table S3*). Personalized virtual clinical scenarios in AR have been found useful in simulating ablation training and guiding catheter navigation. The combination of 3D modelling, simulation, and XR display can also be found in assessing the optimal location and sizing of stents. As a result, the integration of 3D printing technology, simulation, and state-of-the-art virtual and augmented environments opens up new possibilities for the medical field, contributing to more precise diagnoses, more efficient planning, and safer procedures for heart patients.

## Benefits

The implementation of XR in pre-procedural planning and real-time decision-making holds the promise of transforming daily operations in catheterization laboratories. Among the publications cited in this review, the authors indicate a number of significant benefits of XR in improving cardiology procedures.

The main advantage that has been highlighted^28–30,47,49,51,53,57,59,61,63,66,74^ is the possibility of a thorough understanding of the complex heart anatomy. XR have a significant impact on improving the perception of shape and depth. Spatial anatomical orientation provides 3D information about the relationships between structures and increases confidence in treatment strategy planning.^49^ In-depth perception and interactive 3D manipulation in space (zooming, translation and 360-degree rotation of the model) provide unique anatomical views. This may translate into planning patient-specific fluoroscopic projections.^30,37,47,66^ Furthermore, the presentation of data in 3D reduces the mental workload, which speeds up procedural performance while maintaining high-quality procedural effects.^58^ 3D Visualizations in virtual space also support the assessment of procedure feasibility^28,51^ or impact the procedural strategy.^50^ Another advantage of XR is the educational support for trainees or junior operators.^28,30,33^ Virtual clinical scenarios allow for discussing possible complications, presenting methods for optimizing patient recovery. What is more, XR allow remote supervising of subsequent stages of the procedure. An important feature of XR devices is the ability to recognize voice commands,^32,71,72^ which, according to users, ensures sterile working conditions^32^ and provides unlimited access to volumetric data. Thus, it does not interfere with the doctor’s work and is easy to integrate into clinical workflow.^73^ Another important aspect of XR for interventional cardiologists is the possibility of audiovisual communication between experts in real time.^42,50^ Collaboration of multiple users translates not only into the integration of multimodal data in a virtual environment,^28^ but also into the experts’ discussions of treatment solutions.^43^ XR have been assessed as valuable tools for tele-mentoring, supporting operators during procedures.^33^ Visualization of virtual 3D models and dynamic data (virtual catheters, patient records) in real time broadens the spectrum of collaboration between clinical teams.^73^

## Current challenges and technical limitations

Before XR technologies will become part of everyday practice, there are several limitations in their application to overcome in cardiac interventions. Firstly, the quality of 3D visualization strongly depends on the source data provided to the algorithm.^51,54^ Poor data quality can generate errors in the automatic segmentation and rendering steps. Consequently, hardware limitations in processing input data can lead to incorrect visualization of detailed geometries.^40^ Furthermore, the preparation of XR visualizations requires both technological and human resources. Dedicated software, high-performance computers, XR HMD, and technical assistance handling each step of XR visualization preparation are essential.^61^ This involves both equipment and human-generated costs.^73^ Often, XR data preparation needs additional technical and medical experience to properly present valuable data projections, which impacts as well total procedural preparation time. Additionally, XR devices should be integrated with the hospital network and hospital information and archiving systems for better data flow and security. The price of implementation significantly increases the standard XR HMD purchase cost. The IT integration process often requires the support of third parties. Therefore, the XR project may be economically irrelevant for many facilities, especially in low- and middle-income countries. Also, evaluation challenges vary depending on the device’s intended use. For instance, safety and effectiveness concerns differ for preoperative medical image evaluation vs. real-time imaging guidance during procedures. For procedural planning, there is no need for sterile conditions, glasses transparency, or long battery life. However, implementing XR in an operating room setting is more difficult and requires adjustment. The catheterization lab is a demanding environment where electronic devices must withstand temperature swings and radiation. For intraoperative navigation, a secure and stable connection between all XR system components (*Figure 1*) is necessary to eliminate unexpected interruptions during procedure.^8^ For XR hardware and software dedicated to cardiac interventions, there is a gap in validity testing. Currently, there is a lack of guidelines and clinical protocols specific to cardiac interventions regarding the use of XR. In other medical fields, this has been more structured by providing users with instructions for use and clinical scenarios, rating XR usefulness, satisfaction, ease of use, effectiveness, and immersiveness to assess content validity.^80,81^

Frameworks and metrics dedicated to XR technologies evaluation should include both qualitative and quantitative assessment, like subjective operator experience in virtual 3D data perception or direct comparison between linear (2D) and spatial (3D) measurements.

There are also technological obstacles related to intra-procedural XR imaging, such as the inability to render black color,^40^ which can affect data interpretation. Integrating image-guidance into procedures introduces new considerations such as required XR image quality, tracking, usability, and workflow integration. XR-guided procedures pose further evaluation challenges due to real-time tool tracking, often achieved by combining XR devices with stereotaxic. This fusion raises additional hurdles like latency measurement, spatial tool tracking accuracy, and resolving Vergence-Accommodation Conflict.^40^

Another problem with the practical use of XR in the catheterization laboratory is the risk of bias. Single-center studies or tests conducted on a small sample of cases^33,61,66,73^ are not sufficient to generalize the results of the usefulness of XR application. Patients were carefully selected,^66^ and measurements and manipulations on the 3D visualization were often performed postoperatively. The problem of bias also concerns the lack of a blinded trial, the lack a control group, or the lack of randomization in viewing XR models vs. other modalities. Additional information obtained by operators from different imaging modalities at the stage of planning the procedure may distort the conclusions, regarding the benefits of the XR technology itself.^51^ Moreover, operators become more familiar with 3D visualizations, so bias cannot be excluded.

## Future perspectives

Integration of real-time computational simulation based on artificial intelligence (AI) and XR technologies may open a new chapter of patient-tailored cardiac intervention planning.

A promising feature of XR devices is the integration with AI large language models (LLM). Integration of spatial visualization with AI assistant may lead to easier interactions in the XR environment in the future—sharing the field of view on a video call or suggesting useful information regarding presented data.^11^ Procedural scenarios may be matched to the manoeuvres performed by the operator, or XR can support procedure or device-related tips and tricks to the user in real time.^29^ Thanks to AI, virtual guidance systems may compete with standard fluoroscopy in the future, offering the possibility of minimizing nephrotoxic contrast agent and ionizing radiation. Therefore, XR can increase the scope and complexity of procedures performed.^73^

XR also opens the door to clinical content integration and fusion of multimodality imaging. XR Image compilation may eliminate standard angiographic monitors from operating rooms, reducing the equipment in the catheterization laboratory. Removing expensive monitors may impact ergonomics and cost-effectiveness in the future.^73^ To fully exploit the potential of XR in cardiology, continuous improvement of software and hardware is necessary. Automatic segmentation algorithms should include a wider patient population in the studies.^52,73^ More virtual measurement tools, specific for each structure or individual disease, should be advanced as well.^53^ Evaluation of XR devices, including visual and laboratory testing methods, 3D image resolution and contrast, and determination of performance thresholds for intra-procedure tasks, is needed to avoid interobserver variability. Furthermore, XR devices could be supplemented with lenses that would act as radiation shields.^31^ Integration with other head-worn devices (lamps or glasses in the case of surgery) could also expand the application area of XR.^43^ Additional interaction functions and easier access to remote connections without user limits in the virtual space could have a significant impact on the performance of remote consultations, improving education and patient access to specialist care.^39^ Given the increasing use of XR modalities in cardiology, it is essential to create regulatory pathways for the acceptance of XR-based interventions. Single-center, observational studies are the beginning of the next steps. There is a great need for prospective, multicentre, randomized studies^8,66^ to assess the real impact of XR on procedural outcomes. XR models require standardization—studies should validate the performance of critical structures’ measurements in XR compared to measurements in 2D modalities.^43^ The financial feasibility and technical aspects of the widespread adoption of XR in cath labs should also be considered. Cost–benefit analysis must include: implementation costs (device, software, IT infrastructure adoption, dedicated personnel, and time) and benefits (reduced operating time, improved patient outcomes, shorter hospital stay, and reduced care costs). The financial perspective of synthesizing different imaging modalities and integrating XR in the operating room must be examined to see whether the costs of this integration are compensated by the potential added values.^8,73^

## Conclusions

The development of XR opens new opportunities in cardiovascular medicine. XR have a wide range of practical applications from planning interventional procedures, through simulation and combination with technologies such as 3D printing, to intraoperative navigation. Modern medical systems based on XR technologies must meet standard requirements to ensure effective and safe patient service, providing physicians with the highest quality diagnostic tools. Prospective clinical trials involving XR technologies can provide valuable data according to technology safety and effectiveness in real-world clinical scenarios. Valuable research and development of XR technologies in cardiovascular medicine can contribute to more precise, safer, and advanced care for patients.

## Supplementary Material

ztaf072_Supplementary_Data

## Data Availability

The data underlying this article are available in the article and in its online supplementary material.
