# Supplementary material for: Catheterization laboratories open the doors for Extended Realities—review of clinical applications in cardiology
Source: Eur Heart J Digit Health. 2025 Jun 23;6(5):1055–68. doi: 10.1093/ehjdh/ztaf072 (PMC12450514; doi:10.1093/ehjdh/ztaf072)
Supplement: ztaf072_Supplementary_Data [file ztaf072_supplementary_data.docx]

Supplementary Table 1. Use of Extended Realities in preoperative planning - list of cardiac procedures and devices used. Technology: VR – Virtual Reality, AR – Augmented Reality, MR – Mixed Reality; Data: CT- computed tomography, TEE – transoesophageal echocardiography, MRI – magnetic resonance imaging, US - ultrasonography

| **Procedure** | **Technology** | **Data** | **Device, Software** | **No. of patients** | **Type of study** | **References** | **DOI** |
| --- | --- | --- | --- | --- | --- | --- | --- |
| CORONARY | | | | | | | |
| Minimally invasive coronary artery bypass for Kawasaki disease | VR | CT | -, MedicalVR | 1 | Single case | A. H. Sadeghi et al.; European Heart Journal (2020) | /10.1093/eurheartj/ehaa518 |
| Minimally Invasive Coronary Artery Bypass Grafting | VR | CT | - | 17 | Single Center | K. Tachibana et al.; Innovations (2022) | 10.1177/15569845221129212 |
| PCI | VR | - | - | - | Single Center | R. Lal et al.; Heart Lung and Circulation (2020) | 10.1016/j.hlc.2020.09.634 |
| PCI | VR | CT | - | 1 | Single Center | J. Goto et al.; American Journal of Case Reports (2024) | 10.12659/AJCR.944485 |
| ELECTROPHYSIOLOGY | | | | | | | |
| Ablation | VR | CT | HTC Vive, SpectoVR | 1 | Single Case | S. Knecht, et al. Pacing and Clinical Electrophysiology (2019) | 10.1111/pace.13479 |
| Transvenous lead extraction | VR | CT | - | 10 | Single Center | Carretero, et al. Indian Journal of Thoracic and Cardiovascular Surgery (2024) | 10.1007/s12055-023-01663-9 |
| STRUCTURAL | | | | | | | |
| Aortic Dissection | MR | CT | Microsoft HoloLens2, CarnaLife Holo | 1 | Single Case | Tsung-Ying Tsai et al.; Journal of the American College of Cardiology (2024) | 10.1016/j.jacc.2024.04.040 |
| Aortic valve leaflet reconstruction | MR | CT | - | 26 | Single Center | T. Tedoriya et al.; The Journal of the Heart Team (2020) | 10.1080/24748706.2020.1715149 |
| Atrial Septal Defect | VR | TEE | Oculus Rift 2, Medical Imaging XR | 1 | Single Case | A. Zlahoda-Huzior et al.; European Heart Journal Case Report (2022) | 10.1093/ehjcr/ytac477 |
| Atrioventricular valve surgery | VR | TEE | HTC Vive, VR system reated in-house | 15 | Single Center | K. Pushparajah et al.; JTCVS techniques (2021) | 10.1016/j.xjtc.2021.02.044 |
| AVV repair, ventricular septal defect (VSD) closure, double outlet right ventricle (DORV) repair, TAPVD repair/revision | MR | TEE, CT, MRI | -, True3D EchoPixel | 25 | Single Center | J. C. Lu et al.; Journal of the American Society of Echocardiography (2020) | 10.1016/j.echo.2020.02.003 |
| DORV | VR | CT, MRI | - | 10 | Single Center | E. G. Milano et al.; .; European Heart Journal Digital Health (2021) | 10.1093/ehjdh/ztab087 |
| Congenital Heart Disease (CHD) | MR | CT | - , True3D EchoPixel | 12 | Single Center | Sok-Leng Kang et al.; Pediatric Radiology – Technical Innovation (2020) | 10.1007/s00247-020-04740-y |
| CHD | MR | CT | -, True3D EchoPixel | 7 | Single Center | J. Vettukattil et al.; CHEST Congress (2020) | 10.1007/s10554-020-01853-1. |
| CHD, VSD | MR | CT | Microsoft HoloLens, - | 1 | Single Case | H. Brun et al.; European Heart Journal - Cardiovascular Imaging (2019) | 10.1093/ehjci/jey184 |
| Congenital Heart Surgery | VR | MRI | - | 19 | Single Center | T. S. Sorensen et al.; Springer-Verlag (2008) | 10.1007/s00247-008-1032-5 |
| DORV | MR | CT | Microsoft HoloLens, - | 17 | Single Center | W. Ye et al.; Clinical Radiology (2021) | 10.1016/j.crad.2020.10.017 |
| DORV | VR | MRI | - | 6 | Single Center | K. M. Farooqi et al.; Springer Science+Business Media New York (2015) | 10.1007/s00246-015-1244-z |
| DORV | VR | CT, US | MedicalVR workstation, MedicalVR | 5 | Single Center | J. Peek., et al. Interdisciplinary CardioVascular and Thoracic Surgery (2023) | 10.1093/icvts/ivad072 |
| Dynamic Aortic Root Anatomy | VR | CT | -, Vesalius 3D | 1 | Single Case | K. Kamiya et al.; The Annals of Thoracic Surgery (2021) | 10.1016/j.athoracsur.2021.06.038 |
| Left atrial appendage (LAA) closure | VR | CT | Meta Quest 2, VMersive (Warszawa, Poland) | 21 | Single Center | H. Heidari et al.; Frontiers in Cardiovascular Medicine (2023) | 10.3389/fcvm.2023.1188571 |
| LAA closure | VR | CT | Meta Quest 2, Gravity Sketch | 1 | Single Case | T. Shimura, et al. European Heart Journal-Case Reports (2023) | 10.1093/ehjcr/ytad503 |
| LAA occlusion | VR | CT | Meta Quest 2, Mimics Materialise | 10 | Single Center | H. Heidari, et al. Frontiers in Cardiovascular Medicine  (2024) | 10.3389/fcvm.2024.1328906 |
| LAA occlusion | MR | CT | Microsoft HoloLens, - | 4 | Single Center | M. Pasquali et al. Journal of Cardiovascular Computed Tomography (2022) | 10.1016/j.jcct.2022.02.010 |
| LAA occlusion | VR | CT | Vive  D2P® software (3D Systems Inc. Littleton CO, USA) | 20 | Single Center | S. Tejman-Yarden, et al. Journal of Cardiovascular Computed Tomography (2023) | 10.1016/j.heliyon.2023.e14790 |
| LAA occlusion | MR | TEE, CT | -, True3D EchoPixel | 154 | Single Center | J. Deutcher et al.; JACC Journals (2020) | 10.1016/S0735-1097(20)31833-7 |
| Minimally invasive and complex adult cardiac surgery | VR | CT | Oculus Rift S, CardioVR | 6 | Single Center | A. H. Sadeghi et al.; European Heart Journal Digital Health (2020) | 10.1093/ehjdh/ztaa011 |
| Mitral Valve Annuloplasty | VR/MR | TEE | - | 50 | Single Center | J. Ender et al.; Published by Elsevier Inc (2008) | 10.1016/j.athoracsur.2008.07.073 |
| Mitral Valve Disease | MR | TEE | - | 1 | Single Case | C. J. Larotte; Nueva Granada Military University (2019) | 10.1145/3358961.3358991 |
| Mitral valve replacement | VR | CT | Oculus Rift S, - | 1 | Single Case | J. M. Castellanos et al.; European Heart Journal (2022) | 10.1093/ehjcr/ytac384 |
| Paravalvular leakage (PVL) | VR | CT | - , Cardio VR | 6 | Single Center | A. H. Sadeghi et al.; Cardiovascular Interventions (2021) | 10.1016/j.jcin.2021.06.018 |
| Severe mitral regurgitation | MR | TEE | -, True3D EchoPixel | 20 | Single Center | F. Ballocca et al.; Journal of Cardiothoracic and Vascular Anesthesia (2019) | 10.1053/j.jvca.2018.08.013 |
| Sinus Venosus Defects | VR | CT, MRI |  | 28 | Single Center | A. Tandon et al.; JACC: Cardiovascular Imaging (2019) | 10.1016/j.jcmg.2018.10.013 |
| TAVI | MR | CT | -, True3D EchoPixel | 38 | Single Center | K. Sinha et al.; JACC Journals (2019) | 10.1016/j.jcin.2019.01.177 |
| TAVI | VR | CT | Oculus Rift, VisuaMed | 11 | Single Center | X. Ruyra et al.; Interactive CardioVascular and Thiracic Surgery (2022) | 10.1093/icvts/ivac248 |
| TAVI | VR | CT | - | 60 | Single Center | D. Kanschik et al.; JAHA (2024) | 10.1161/JAHA.123.034086 |
| ‍TAVI | MR | CT | - ,  ARTICOR®, Artiness srl, Mediolan | 100 | Single Center | M. Bonanni et al.; International Journal of Cardiology (2024) | 10.1016/j.ijcard.2024.132330 |
| Transcatheter pulmonary valve replacement | VR | CT | - , Elucis VR | 1 | Single Case | A. Salavitabar, Journal of the Society for Cardiovascular Angiography & Interventions (2024) | 10.1016/j.jscai.2024.101302 |
| Ventricular Septal Defect Evaluation | VR | MRI | - | 1 | Single Case | A. Mendez et al.; Cardiovascular flashlight (2018) | 10.1093/eurheartj/ehy685 |
| Venus P-valve implantation | MR | CT | Microsoft HoloLens (Magic Leap), Artiness  (Milan, Italy), | 17 | Single Center | A. D’Aiello et al.; Frontiers in Cardiovascular Medicine (2024) | 10.3389/fcvm.2024.1378924 |

Supplementary Table 2. Use of Extended Realities in intraprocedural monitoring - list of cardiac procedures and devices used. Technology: VR – Virtual Reality, AR – Augmented Reality, MR – Mixed Reality; Data: CT- computed tomography, TEE – transoesophageal echocardiography, MRI – magnetic resonance imaging, US – ultrasonography.

| **Procedure** | **Technology** | **Data** | **Device, Software** | **No. of patients** | **Type of study‍** | **References** | **DOI** |
| --- | --- | --- | --- | --- | --- | --- | --- |
| CORONARY | | | | | | | |
| PCI | MR | CT | Microsoft HoloLens 2, CarnaLife Holo | 1 | ‍Single Case | A. Złahoda-Huzior, et al.; European Heart Journal, Cardiovascular Imaging (2025) | 10.1093/ehjci/jeae337 |
| PCI chronic total occlusion RCA | AR | CT | Google Glass, - | 1 | ‍Single Case | M. Opolski, et al.; Canadian Journal of Cardiology (2016) | 10.1016/j.cjca.2015.08.009 |
| ‍Remote proctoring PCI CTO | MR | - | Microsoft HoloLens 2 | 10 | ‍Single Center | S. Calic, et al. ; EHJ Digital Health (2024) | 10.1093/ehjdh/ztae037 |
| Revascularization of Coronary Chronic Total Occlusion | AR | CT | Google Glass, - | 15 | ‍Single Center | M. Opolski et al.; Journal of Cardiovascular Computed Tomography (2017) | 10.1016/j.jcct.2017.09.013 |
| ELECTROPHYSIOLOGY | | | | | | | |
| Cardiac resynchronization therapy | AR | CT | Microsoft HoloLen, CarnaLife | 1 | Single Case | M. Witkowski, et al. Polish Heart Journal (2019) | 10.33963/KP.14801 |
| Navigational mapping | MR | - | Microsoft HoloLen, SentiAR | 16 | Single Center | D. Bloom et al. Cardiovascular Digital Health Journal (2023) | 10.1016/j.cvdhj.2023.06.003 |
| Peacemaker implantation | AR | CT | Google Glass, - | 1 | ‍Single Case | M. Opolski et al.; Interventional Cardiology (2018) | 10.5603/CJ.2018.0058 |
| Transcatheter cardiac mapping and ablation | MR | Cardiac mapping | Microsoft HoloLens, SentEP SentiAR | 16 | Single Center | J. N. Avari Silva et al.; JACC Clin Electrophysiol. (2021) | 10.1016/j.jacep.2020.04.036 |
| Transcatheter cardiac mapping and ablation | MR | Cardiac mapping | Microsoft HoloLens, ĒLVIS | 10 | Single Center | M. K. Southworth et al.; IEEE Journal of Translational Engineering in Health and Medicine (2020) | 10.1109/JTEHM.2020.3007031 |
| STRUCTURAL | | | | | | | |
| Balloon mitral commissurotomy | MR | TEE | Microsoft HoloLens 1, CarnaLife Holo | 1 | ‍Single Case | J. D. Kasprzak et al.; European Society of Cardiology (2020) | 10.1093/eurheartj/ehz127 |
| Cogenital heart disease | MR | CT | Microsoft HoloLens, - | 1 | ‍Single Case | H. Brun et al.; European Heart Journal: Cardiovascular Imaging (2019) | 10.1093/ehjci/jey184 |
| ‍Guidance for Cerebral Embolic Protection During TAVI | AR | CT, MRI, TTE | - | 24 (12) | Single Center | S. Sadri, et al.; JACC (2024) | 10.1016/j.jacadv.2024.100839 |
| Guidance navigation for beating heart mitral valve repair | MR+3DP | CT | Microsoft HoloLens, - | 1 | ‍Single Case | G. Butera et al.; JACC: Cardiovascular Interventions (2020) | 10.1016/j.jcin.2019.03.020 |
| Guide percutaneous transcatheter ASD closure and pulmonary valve implantation | VR/MR | TEE,  angio-CT | - , Realview Imaging Inc | 8 | Single Center | E. Bruckheimer et al.; European Heart Journal: Cardiovascular Imaging (2016) | 10.1093/ehjci/jew087 |
| Inferior vena cava filter implantation | MR | CT | Microsoft HoloLens, - | 1 | ‍Single Case | H. Zhu et al.; J Geriatr Cardiol. (2019) | 10.11909/j.issn.1671-5411.2019.07.008 |
| LAA occlusion | MR | CT | Microsoft HoloLens 2, CarnaLife Holo | 2 | Single Center | Zbroński, Karol, et al Polish Heart Journal (Kardiologia Polska) (2018) | 10.5603/KP.2018.0017 |
| MitraClip Implantation‍ | AR | 3D USG | Microsoft HoloLens 2, CarnaLife Holo | 1 | ‍Single Case | J. Sacha, et al.; JACC: Cardiovascular Interventions (2022) | 10.1016/j.jcin.2022.01.023 |
| Percutaneous patent ductus arteriosus closure | MR | CT | Microsoft HoloLens 1, CarnaLife Holo | 1 | ‍Single Case | J. D. Kasprzak et al.; European Society of Cardiology (2019) | 10.1093/ehjci/jez008 |
| Remote proctoring during structural heart intervention‍ | MR | - | - | 9 | Single Center‍ | G. Ascione et al; Cathether Cardiovasc Interv. (2024) | 10.1002/ccd.31187 |
| Right Heart Catherization | MR | CT, ICA | - | 282 | Single Center | J. Chahine et al. Cardiovascular Revascularization Medicine (2022) | 10.1016/j.carrev.2022.08.009 |
| Single-stage unifocalisation for pulmonary atresia (PA) with VSD and major aortopulmonary collateral arteries (MAPCA) | VR/MR | CT | Microsoft HoloLens, - | 5 | Single Center | J. Cen et al.; Heart: Lung and Circulation (2020) | 10.1016/j.hlc.2020.03.017 |
| TAVI | AR | CT | Microsoft HoloLens, - | 6 | Single Center | S. Sadri et al.; Circulation (2018) | 10.1161/circ.138.suppl_1.12019 |
| TAVI | MR | CT, MRI | Microsoft HoloLens 1, CarnaLife Holo | 1 | ‍Single Case | B. Rymuza et al.; Kardiologia Polska (2017) | 10.5603/KP.2017.0195 |
| TAVI | MR | USG | Microsoft HoloLens, - | 9 | Single Center | M. Alonso-Felipe, et al. Journal of Healthcare Informatics Research (2023) | 10.1007/s41666-023-00147-0 |
| TAVI | AR | TEE | - , - | 10 | Single Center | M. E. Currie et al.; Sage Journals Innovations: Technology and Techniques in Cardiothoracic and Vascular Surgery (2016) | 10.1097/imi.0000000000000235 |
| Use of US for vascular access | MR | US | Microsoft HoloLens, MantUS SentiAR | - | Single Center | D. Bloom et al.; Journal of the American College of Cardiology (2022) | 10.1016/S0735-1097(22)03027-3 |

Supplementary Table 3. Use of Extended Realities with other technologies.

| **Procedure** | **Technology** | **Combined technology** | **Purpose** | **Type of study‍** | **Reference** | **DOI** |
| --- | --- | --- | --- | --- | --- | --- |
| Ablation | AR | Simulation | to evaluate the potential impact of catheter navigation accuracy improvement | Single Center | A. Prakosa, et al.; Computers in biology and medicine (2021) | 10.1016/j.compbiomed.2021.104366 |
| Aortic coarctation | VR | Simulation | to assess whether simulations of stent implantation in VR can be used for planning intravascular treatment of aortic coarctation | Single center‍ | N. Topuzov, et al. Advances in Interventional Cardiology (2022) | 10.5114/aic.2022.120377 |
| Transcatheter Cardiac Mapping and Ablation | MR | Simulation | to simulate a representative high load scenario | Performance evaluation study‍ | MK Southworth et al. IEEE J Transl Eng Health Med. (2020) | 10.1109/JTEHM.2020.3007031 |
| TAVI | AR | Simulation | to show the catheter approach the target deployment depth marked by target plane | Single center‍ | ME Currie et al. Innovations (Phila) (2016) | 10.1097/imi.0000000000000235 |
| Paravalvular leak (PVL) after a TAVI | VR | Simulation | to predicted the presence and absence of PVL after TAVI procedure | Single center‍ | J. Chahine et al. Journal of Invasive Cardiology (2024) | 10.25270/jic/24.00019 |
| PCI | VR | Simulation | to solve the catheter approach | Single Case | H. Higami, et al. European Heart Journal-Case Reports  (2023) | 10.1093/ehjcr/ytad507 |
| PCI | VR | Simulation | to provide a suitable platform for PCI surgery training and rehearsal | Single Center | L. Shuai, et al. IEEE Transactions on Visualization and Computer Graphics (2021) | 10.1109/TVCG.2021.3106478 |
| Left atrial appendage closure | VR | Simulation | to visualize the ideal delivery sheath curve for alignment with the patient’s left atrial appendage anatomy | Single case | F. Sawada, et al. European Heart Journal-Case Reports (2024) | 10.1093/ehjcr/ytae399 |
| Left atrial appendage occluder interventions | VR | 3D printing, Simulation | improve the planning of LAAO interventions | Performance evaluation study‍ | J. Mill et al.; International journal of bioprinting (2022) | 10.18063/ijb.v9i1.640 |
